# [Expression of Concern] Establishment and evaluation of a new highly metastatic tumor cell line 5a-D-Luc-ZsGreen expressing both luciferase and green fluorescent protein

**DOI:** 10.3892/ijo.2025.5840

**Published:** 2025-12-17

**Authors:** Hitomi Sudo, Atsushi B. Tsuji, Aya Sugyo, Hiroyuki Takuwa, Kazuto Masamoto, Yutaka Tomita, Norihiro Suzuki, Takeshi Imamura, Mitsuru Koizumi, Tsuneo Saga

Int J Oncol 48: 525-532, 2016; DOI: 10.3892/ijo.2015.3300

Following the publication of the above paper, it was drawn to the Editor's attention by a concerned reader that, regarding the bioluminescence and fluorescence images of the mouse shown in Fig. 2C and E respectively, the images/positioning of the mouse appeared to be unexpectedly similar, and the authors were asked to confirm whether the images were captured at the same time, but separate results were obtained for the luminescence and fluorescence readings. Similarly, in Fig. 3A, the same mouse image (re. its appearance and positioning) appeared to be shown for the 5a-D-Luc cell line on Days 14 and 28, albeit with different bioluminescence. Finally, upon analyzing the data independently in the Editorial Office, it came to light that, for the phase contrast images of the MDA-MD-231 and 5a-D-Luc cell lines shown in Fig. 1A, these appeared to be the same image, but with different lighting intensities and with the 5a-D-Luc image rotated through 90°.

The authors were contacted by the Editorial Office to offer an explanation for these possible anomalies in the presentation of the data in this paper, although up to this time, no response from them has been forthcoming. Owing to the fact that the Editorial Office has been made aware of potential issues surrounding the scientific integrity of this paper, we are issuing an Expression of Concern to notify readers of this potential problem while the Editorial Office conitnues to investigate this matter further.

